# In Vitro Antiparasitic Activities of Monovalent Ionophore Compounds for Human and Canine Leishmaniases

**DOI:** 10.3390/ani12182337

**Published:** 2022-09-08

**Authors:** Estefanía Calvo Alvarez, Sarah D’Alessandro, Daniela Proverbio, Eva Spada, Roberta Perego, Donatella Taramelli, Nicoletta Basilico, Silvia Parapini

**Affiliations:** 1 Dipartimento di Scienze Biomediche, Chirurgiche e Odontoiatriche, Università degli Studi di Milano, Via Pascal, 36, 20133 Milan, Italy; 2 Dipartimento di Scienze Farmacologiche e Biomolecolari, Università degli Studi di Milano, Via Pascal, 36, 20133 Milan, Italy; 3 Dipartimento di Medicina Veterinaria e Scienze Animali, Università degli Studi di Milano, Via dell’Università, 1, 26900 Lodi, Italy; 4 Dipartimento di Scienze Biomediche per la Salute, Università degli Studi di Milano, Via Pascal, 36, 20133 Milan, Italy

**Keywords:** ionophores, *Leishmania* spp., in vitro activity, canine/human macrophages, dog

## Abstract

**Simple Summary:**

The leishmaniases are vector-borne, neglected diseases caused by parasites of the genus *Leishmania*, widely diffused around the globe. Clinical manifestations range from localized cutaneous and mucocutaneous lesions to the visceral form, with potentially fatal outcomes. Associated with malnutrition, a weak immune system, and no access to health-care facilities, these conditions affect the poorest populations worldwide. In addition, dogs develop a multisystemic and fatal disease, and act as the main parasite reservoir for some *Leishmania* species. Currently, human, and canine leishmaniases share the same treatment, which includes very few, difficult to administer, expensive, and toxic drugs. Moreover, drug resistance is increasingly spreading, and there is no human vaccine. Therefore, improved and safer treatments are a high priority. Here, monovalent ionophores (salinomycin, monensin, and nigericin) were repurposed in vitro for their leishmanicidal abilities against both insect-stage parasites (promastigotes) and intracellular forms (amastigotes) within human and primary canine macrophages. These compounds showed similar antiparasitic effects against distinct *Leishmania* spp. promastigotes. Interestingly, a differential and host-specific anti-amastigote efficacy was observed, being these compounds more active against human- than canine-infected macrophages. Altogether, these data indicate a potential application of ionophores against *Leishmania* infections and challenge the concept that the same compounds may be equally effective against both human and canine leishmaniases.

**Abstract:**

The leishmaniases are vector-borne parasitic diseases affecting humans and animals, with high mortality rates in endemic countries. Infected dogs represent the main reservoir of infection. Disease control is mainly based on chemotherapy, which, at present, shows serious drawbacks both in humans and dogs. Therefore, the discovery or repurposing of new treatments is mandatory. Here, three monovalent ionophores (salinomycin, monensin, nigericin) were tested against promastigotes of *Leishmania (L.) infantum*, *Leishmania tropica*, and *Leishmania braziliensis*, and against amastigotes of *L. infantum* within human and, for the first time, canine macrophages. All three drugs were leishmanicidal against all *Leishmania* spp. promastigotes with IC_50_ values between 7.98 and 0.23 µM. Monensin and nigericin showed IC_50_ values < 1 µM, whereas salinomycin was the least active compound (IC_50_ > 4 µM). Notably, the ionophores killed *L. infantum* amastigotes within human THP-1 cells with IC_50_ values ranging from 1.67 to 1.93 µM, but they only reduced by 27–37% the parasite burden in *L. infantum*-infected canine macrophages, showing a host-specific efficacy. Moreover, a selective higher toxicity against canine macrophages was observed. Overall, repurposed ionophores have the potential to be further investigated as anti-*Leishmania* agents, but different drug options may be required to tackle human or canine leishmaniases.

## 1. Introduction

Leishmaniases are a group of complex vector-borne diseases caused by the infection with the protozoan parasites of the genus *Leishmania*, transmitted to humans and animals by the bite of female phlebotomine sand flies. In humans, more than 20 species of *Leishmania* are responsible for three main forms of the disease, which depend on the parasite species involved and the host immune response [1]. The most common is the cutaneous leishmaniasis (CL), the most disabling is the mucocutaneous leishmaniasis (MCL), and the most severe is the visceral leishmaniasis (VL), also known as kala-azar, which is potentially fatal in the absence of treatment [2]. Indeed, the World Health Organization estimates 30,000 new cases of VL and more than 1 million new cases of CL annually, with approximately 20,000–40,000 deaths per year [3]. The disease is prevalent in the tropics, subtropics, and southern Europe, and is classified as a neglected tropical disease (NTD), affecting the world’s poorest populations while trapping them in a long-lasting cycle of poverty and illness.

Although the parasite can infect about 70 species of mammals, natural infections in rodents and canids are the most common. In particular, *Leishmania (L.) infantum*, which is the causative agent of canine leishmaniasis (CanL), is also responsible for a zoonotic form of the disease, human visceral leishmaniasis, where the domestic dog is the main parasite reservoir [4,5].

Despite huge efforts in the past decades, there is still no human vaccine and, although vaccines to prevent CanL are currently available, doubts regarding their efficacy exist [6]. Therefore, chemotherapy remains the most effective strategy, with common drugs being used to treat CanL and human leishmaniasis, including: antimonials, amphotericin B, miltefosine, allopurinol, and paromomycin, either as monotherapy or in drug combinations [7]. However, these few existing drugs present many disadvantages such as relapses, high toxicity, and the emergence of drug resistance. In addition, sustained transmission can also occur after treatment, mainly when treated dogs continue to act as reservoirs for human infection in the case of incomplete parasitological cure [8]. Accordingly, the identification of novel compounds is vital. To this end, drug repositioning (i.e., the usage of existing drugs for new therapeutic indications) represents an attractive approach to significantly reduce development costs, time-to-market, and risks of failure. This is particularly appropriate for diseases of developing countries like the leishmaniases, for which industrial R&D (Research and Development) and market economical returns are very limited [9].

Polyether antibiotics or carboxyl ionophores are a unique class of molecules with outstanding potency against critical infections caused by bacteria, viruses, and parasites [10]. Among them, salinomycin is a monovalent ionophore for alkali ions with relative K+ selectivity, thus able to interfere with mitochondrial functions [11]. Patented as an anticoccidial agent in 1974, salinomycin has been used in livestock since. Salinomycin and other ionophores such as monensin and nigericin, which are sodium and potassium antiporters, respectively, were also found to inhibit cancer stem cell growth by different mechanisms [12,13]. In addition, these monovalent ionophores have also demonstrated broad antiparasitic activity both in vitro and in vivo against different unicellular or multicellular pathogens [14], including *Plasmodium falciparum*, as shown by our research group [15]. Regarding the leishmaniases, it has been reported that salinomycin is active against promastigotes of *Leishmania (L.) donovani* by interacting with plasma membrane lipids and reducing ATP production [16]. Furthermore, monensin has showed in vitro, ex vivo, and in vivo anti-leishmanial activities on amastigotes of *L. donovani* and *Leishmania major* [17,18].

Beyond human-infecting *Leishmania* spp. and human target cells, data on the activity of the ionophore compounds against intracellular *Leishmania* amastigotes within primary canine macrophages are still missing. Therefore, the aim of the present work was to first test the activity of monensin, salynomicin, and nigericin against extracellular promastigotes of *L. tropica*, *L. braziliensis*, and *L. infantum*, serving as etiological representatives of human CL, MCL, and VL. Subsequently, the development of an in vitro model to infect primary canine macrophages with *L. infantum* permitted us to assess the unknown antiparasitic activities of such ionophores against intra-macrophage amastigotes, and further compare the results with that of its human counterparts.

## 2. Materials and Methods

### 2.1. Human Cells and Leishmania spp. Promastigote Cultures

THP-1 cells (human acute monocytic leukemia cell line) were maintained in Roswell Park Memorial Institute (RPMI) 1640 medium supplemented with 10% fetal bovine serum (FBS, EuroClone), 50 µM 2-mercaptoethanol, 20 mM HEPES, and 2 mM L-glutamine, at 37 °C in 5% CO_2_.

Promastigote stages of *L. infantum* strain (MHOM/TN/80/IPT1) (WHO international reference strain, kindly provided by Dr. M. Gramiccia and Dr. T. Di Muccio, ISS, Roma, Italy), and clinical isolates of *L. tropica* (MHOM/SY/2012/ISS3130) and *L. braziliensis* (MHOM/PE/2006/ISS2848) (kindly provided by Dr. R. Grande, Sacco Hospital, Milan, Italy), were cultured in Schneider’s Drosophila medium (Lonza) supplemented with 2 mM L-glutamine and 10% heat-inactivated FBS (HyClone) at 24 °C.

### 2.2. Isolation of Canine Peripheral Blood Mononuclear Cells

Blood collected from healthy blood donor dogs (*n* = 12) of different breeds, middle age, and both sexes, was used. The blood was collected during routine blood sampling for annual control. According to the University of Milan animal use regulations, formal ethical approval was not needed as dogs were sampled with the informed consent of the owners during routine visits for prophylactic reasons, and the owners gave their consent for the use of excess blood after routine testing in further studies (EC decision 29 October 2012, renewed with the protocol n° 02-2016). Peripheral blood mononuclear cells (PBMC) were isolated from heparinized whole blood samples (10 mL each) through density gradient centrifugation. Briefly, the whole blood volume was diluted in RPMI 1640 at 1:1 ratio and overlaid on Ficoll-Hypaque (Amersham Biosciences, UK; density: 1.119 g/mL) at 2:1 Ficoll:blood ratio. All samples were centrifuged at 1700 rpm for 30 min at room temperature. The PBMC ring was collected at the interface and transferred to another tube with 30 mL of RPMI-1640, which was centrifuged twice at 1700 rpm for 7 min at room temperature. Platelets were removed by mild centrifugation at 900 rpm for 7 min. Isolated PBMC were resuspended in RPMI-1640 supplemented with 10% FBS, 2 mM L-glutamine, and 20 mM HEPES buffer. Cells were plated at a density of 2 × 10^5^ cells in Lab-Tek chamber slides (Nunc) and allowed to adhere for 2 h at 37 °C and 5% CO_2_. Cells were then washed twice with phosphate buffer saline (PBS) to remove non-adherent cells, and the differentiation protocol was initiated.

### 2.3. Cytotoxicity and Leishmania Promastigote Viability Assay

To estimate the 50% inhibitory concentration (IC_50_) of compounds on both cells and *Leishmania* promastigotes, the MTT (3-[4,5-dimethylthiazol-2-yl]-2,5-diphenyltetrazolium bromide) assay was used with minor modifications [19,20]. Briefly, monensin and nigericin were dissolved in ethanol (10 mg/mL), and salinomycin and amphotericin B in DMSO (10 mg/mL). All compounds were then diluted with medium to achieve the required concentrations (final DMSO or ethanol concentration <1%, non-toxic to the parasite or cells). The complete medium used for the antileishmanial activity assay was RPMI 1640 (EuroClone) supplemented with 10% heat-inactivated FBS (EuroClone), 20 mM HEPES, and 2 mM L -glutamine. Drugs were added to 96-well round-bottom microplates and seven serial dilutions were performed. Amphotericin B was used as the reference anti-*Leishmania* drug. Subsequently, 100 μL of the parasite suspension (5 × 10^6^ parasites/mL) was dispensed into the plates. After 72h of incubation at 24 °C, 20 μL of MTT solution (5 mg/mL in PBS) was added into each well. After 3 h in the dark, the plates were centrifuged, the supernatants discarded, and the formazan crystals in the pellet dissolved in 100 μL of lysing buffer (20% *w*/*v* of a solution of SDS -Merck-, and 40% of N,N-dimethylformamide -Merck- in H_2_O).

For cell cytotoxicity experiments, 100 μL (5 × 10 ^5^ cells/mL) of THP-1 cells were plated in 96-well flat-bottom microplates and treated with 0.1 µM phorbol myristate acetate (PMA, Merck, Kenilworth, NJ, USA) for 72 h to achieve differentiation into macrophages. Cells were then treated with different drug concentrations (60–0.4 µM). After 72 h, 20 μL of the MTT solution was added into each well. After 3 h in the dark, the supernatants were discarded and the formazan crystals dissolved in 100 μL of the same lysing buffer described above. The absorbance was then measured spectrophotometrically at 550 nm (reference wavelength 650 nm). The results are expressed as IC_50_, which is the dose of compound necessary to inhibit parasite and cell growth by 50%. The IC_50_ values were extrapolated from nonlinear regression analysis of the concentration–response curve using the software Gen5 1.10 provided with the Synergy 4 (BioTek, Winooski, VT, USA) reader. Data are the mean ± standard deviation of individual experiments performed in duplicate.

### 2.4. In Vitro Differentiation of Human and Canine Macrophages and Intracellular Amastigote Susceptibility Assays

For *Leishmania* spp. infections, 100 μL of THP-1 cells (5 × 10^5^ cells/mL) or canine macrophages (2 × 10^6^ cells/mL) were plated in 16-chamber Lab-Tek culture slides (Nunc) and further treated with 0.1 µM of PMA for 72 h to achieve differentiation into macrophages [21]. Cells were then washed with PBS and infected with stationary phase *L. infantum* promastigotes at a macrophage:promastigote ratio of 1:10. After 24 h, cell monolayers were extensively washed with PBS to remove non-internalized promastigotes and observed under an inverted light microscope to evaluate both the cellular size and the cellular morphology. Infected cells were then incubated in the presence of test compounds for 72 h. Infected and/or treated cells were then fixed with 100% methanol and stained with Giemsa at room temperature. The percentage of infected macrophages in treated and non-treated wells was determined by counting 200–300 macrophages/well under a light microscope. About 10 random microscopic fields/well were observed. The number of infected macrophages in the untreated control samples was considered 100% for calculating the percentage of infection in drug treated samples. IC_50_ values were then calculated. Data are the mean ± standard deviation of individual experiments performed in triplicate.

## 3. Results

### 3.1. In Vitro Activity of Ionophores against L. infantum, L. tropica and L. braziliensis Promastigotes

**Figure 1:**
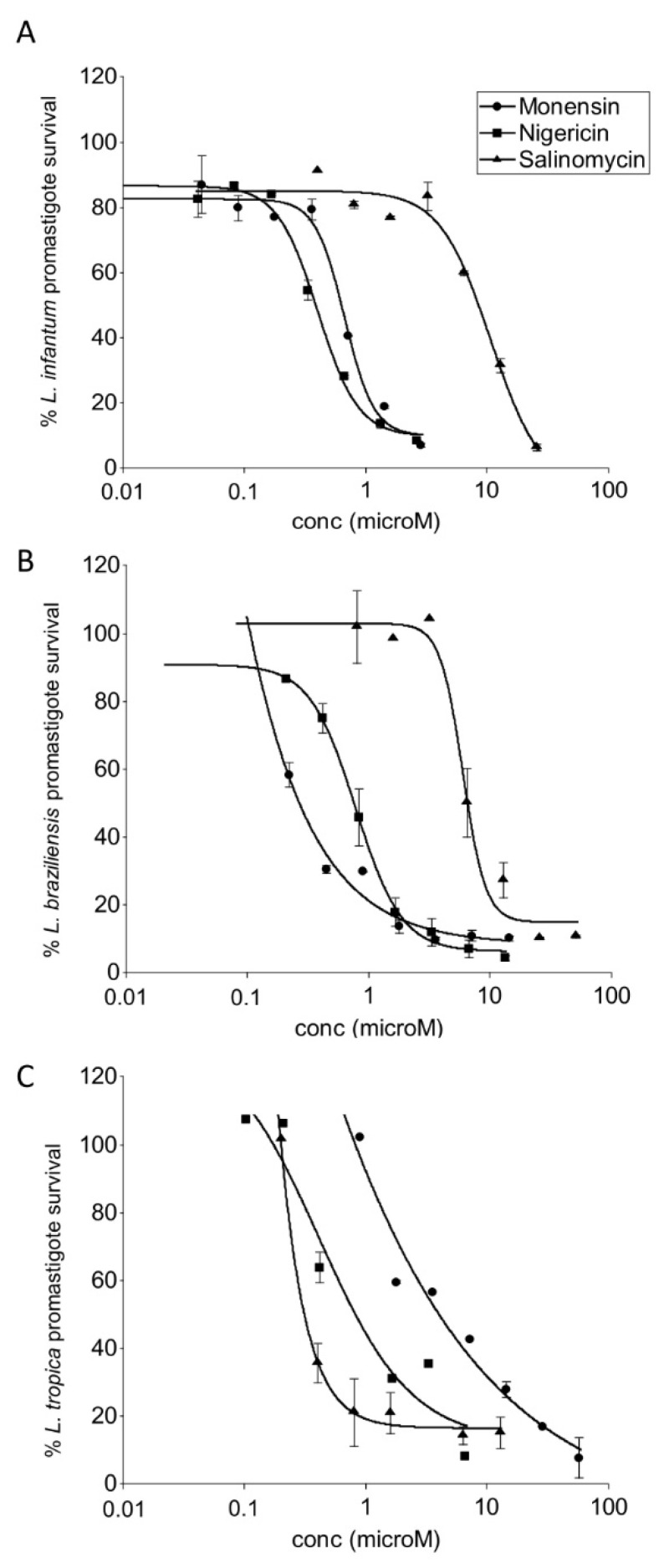
Dose-response curves of the effect of salinomycin, monensin, and nigericin on the growth of promastigotes of *L. infantum* (**A**), *L. braziliensis* (**B**), and *L. tropica* (**C**). Each point represents the mean ± SD from one representative experiment in duplicate out of three conducted in the same conditions.

Salinomycin, monensin, and nigericin were tested in vitro against promastigotes of *L. infantum*, *L. tropica*, and *L. braziliensis*, the causative agents of VL, CL and MCL, respectively. A dose-dependent antileishmanial activity was observed for all three compounds (Figure 1). Monensin and nigericin exhibited comparable activities with IC_50_s < 1 µM against all *Leishmania* promastigote species tested. In contrast, salinomycin was the least active compound with an IC_50_ higher than 4 µM (Table 1). Amphotericin B, a currently used drug for leishmaniasis [5], confirmed its strong potency.

### 3.2. In Vitro Cytotoxicity of Ionophores against Human Cells and Antiparasitic Activity against Intracellular Amastigotes of L. infantum

Non-infected human macrophages (PMA-differentiated THP-1, dTHP-1) and human dermal fibroblasts (HDF) were incubated in the presence of different concentrations of ionophores to determine the nontoxic concentration for mammalian cells. As shown in Table 2, none of the tested ionophores were cytotoxic to HDF at doses as high as 60 μM. Monensin and nigericin exhibited non-toxic effects against dTHP-1, whereas salinomycin showed a mean CC_50_ of 32.1 μM (Table 2).

Next, human macrophages (dTHP-1) were infected with stationary-phase promastigotes of *L. infantum* at a cell:promastigote ratio of 1:10 for 24 h, and allowed to differentiate into intracellular parasite forms. After the removal of free promastigotes, the infected macrophages were incubated for 72 h in the presence of the ionophores at different concentrations. A significant reduction in the number of infected macrophages was observed after treatment with all the compounds tested. The inhibition was dose-dependent with IC_50s_ between 1 and 2 µM (Table 3). Interestingly, all the compounds showed a strong selectivity against *L. infantum* parasites, as confirmed by the high selectivity index (SI), calculated as the ratio between cytotoxicity (CC_50_) and activity (IC_50_) against *Leishmania* (Table 3).

### 3.3. L. infantum and L. tropica Infection of Canine Macrophages

**Figure 2:**
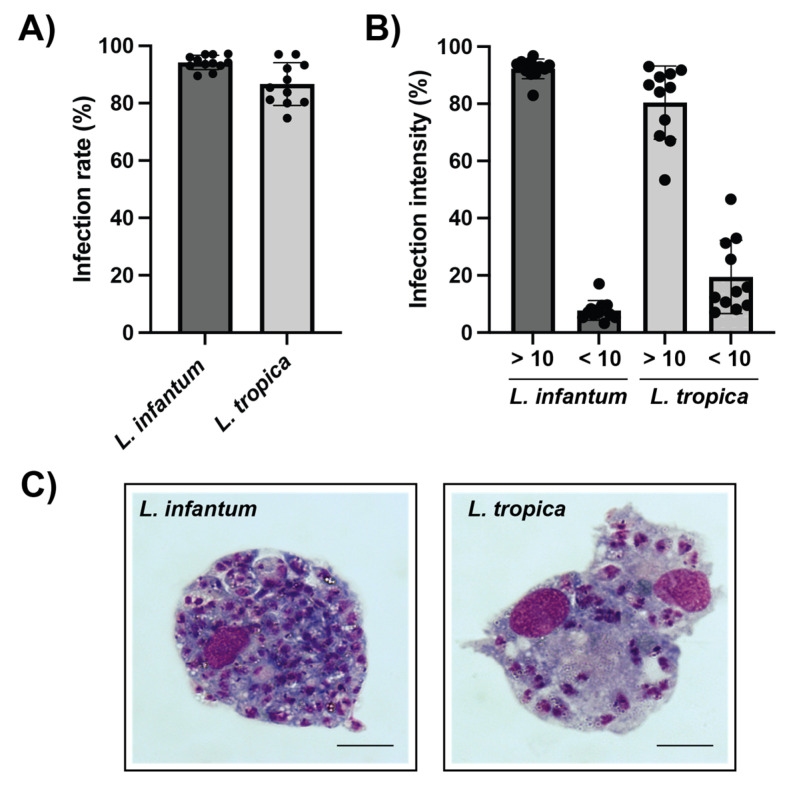
*Leishmania* spp. infection of differentiated primary canine macrophages. (**A**) Upon ex vivo isolation from whole peripheral blood and further differentiation with PMA, canine monocyte-derived macrophages were infected with *L. infantum* or *L. tropica* promastigotes during 72 h at a ratio of 1:10 cells:parasites. The proportion of parasite-bearing cells was quantified through Giemsa staining. (**B**) The intensity of the infection was evaluated by counting the number of intracellular amastigotes within infected macrophages. (**C**) Representative optical images of Giemsa-stained canine macrophages containing *L. infantum* (left) and *L. tropica* (right) amastigotes. Note the high number of *L. infantum* intracellular stages within canine cells. Scale bar 5 µm. Data (mean ± SD) from four independent experiments are shown.

**Figure 3:**
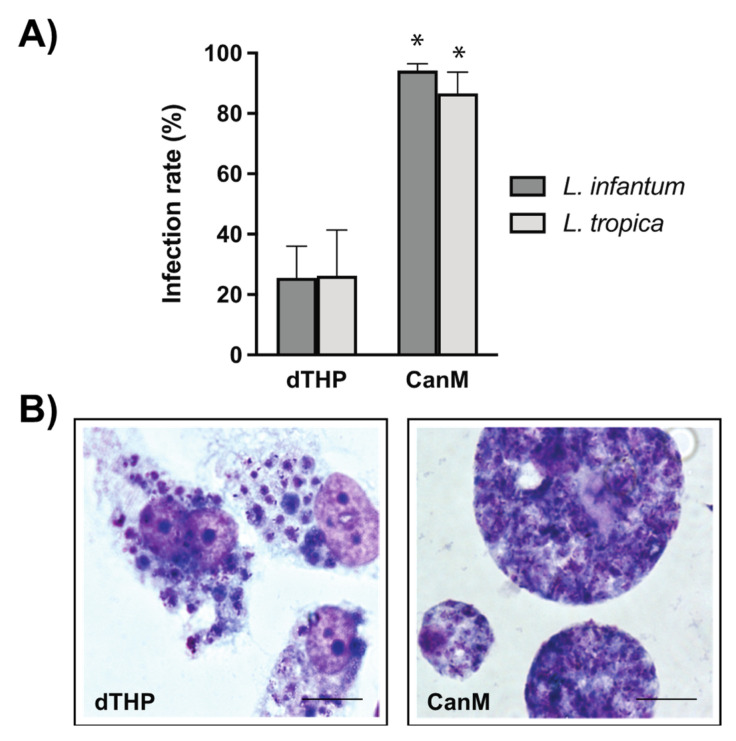
Comparative parasite infection of human vs. canine macrophages. (**A**) Percentage of infected dTHP-1 or canine macrophages (CanM) with *L. infantum* and *L. tropica* promastigotes. Infected cells were Giemsa stained and the percentage of infected cells was calculated. Data are the mean of IC_50_ (µM) ± SD of at least four different experiments in duplicate. Significant differences were found between the infection rates of human and canine macrophages with *L. infantum* and *L. tropica* (* *p* < 0.05, two-way ANOVA and Sidak’s multiple comparison test). (**B**) Selected images of Giemsa-stained dTHP-1 and CanM containing *L. infantum* amastigotes.

Canine vs. human host macrophages were allowed to phagocytose *L. infantum* and *L. tropica* promastigotes, and the potential differences in terms of infection parameters were further investigated. Results indicate that PMA-differentiated canine macrophages heavily phagocytize both *L. infantum* and *L. tropica* promastigotes. At a parasite-to-macrophage ratio of 10:1, the infection rate reached 94.17 ± 2.34% and 86.67 ± 6.96% of *L. infantum*- and *L. tropica*-infected canine macrophages, respectively (Figure 2A and Figure 3A). The intensity of the infection was also very high, with 92% of macrophages infected with *L. infantum* and 80% of *L. tropica*-infected cells containing more than 10 intracellular parasites per macrophage (Figure 2B,C). In contrast, the percentage of infected human dTHP-1 cells was significantly lower (25.5 ± 10.5% and 26.2 ± 15.2% for *L. infantum*- and *L. tropica*-infected dTHP-1, respectively) than that of canine macrophages (Figure 3A).

### 3.4. Anti-Leishmania In Vitro Activity of Ionophores against Intracellular L. infantum Amastigotes in Canine Macrophages

**Figure 4:**
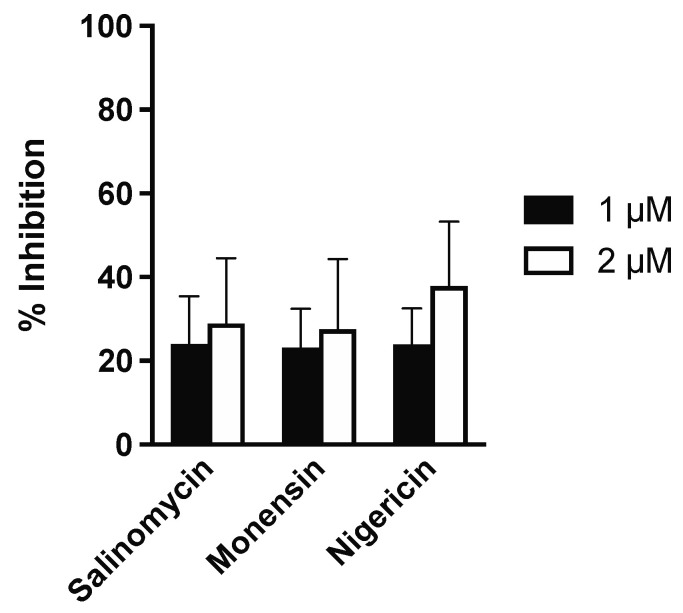
Antiparasitic activity of ionophores against amastigotes of *L. infantum* in canine macrophages. Infected cells were treated with two ionophore concentrations (1 and 2 µM) for 72 h and subsequently stained with Giemsa to calculate the proportion of infected cells. The % of inhibition in drug-treated samples was then quantified by considering the infection in untreated controls as 100%.

Finally, in order to assess the antiparasitic effects of the ionophores against *L. infantum* amastigotes within canine macrophages, freshly-isolated and differentiated primary cells were infected with *L. infantum* promastigotes for 24 h and subsequently treated with different ionophore concentrations, selected according to the observed activity on infected dTHP-1 cells. Notably, none of the tested doses reduced the proportion of infected canine macrophages by more than 50%. The inhibition of *L. infantum* infection was investigated at 1 μM and 2 μM concentrations only (Figure 4), since higher doses resulted toxic to canine macrophages. Specifically, the inhibition of canine macrophage viability at 4 μM was 41.7 ± 13.3, 70.6 ± 7.6 and 68.9 ± 4.2 for salinomycin, monensin, and nigericin, respectively.

## 4. Discussion

Currently available drugs for the treatment of human leishmaniases are quite few and often show toxicity, parasite resistance, and high costs, especially for patients from tropical endemic countries. Similarly, the drugs used to treat *Leishmania* infections in dogs are limited, do not achieve a complete cure of the disease, and can cause important side effects [22]. To potentially increase the number of antileishmanial compounds to treat both human and canine leishmaniases, here we evaluated the activity of different monovalent ionophores (salynomycin, monensin, and nigericin) against three *Leishmania* spp. as causative agents of CL, MCL, and VL. The antiparasitic potential of the ionophores was investigated both in the promastigote stage and against disease-relevant amastigotes of *L. infantum* in human and canine macrophages.

Ionophores are compounds able to bind metal cations and transport them across cell membranes, leading to changes in the sodium/potassium gradient, increased osmotic pressure causing swelling, vacuolization, mitochondrial injuries, and cell death [23]. Several ionophores have shown promising in vitro and in vivo activities against the causative agents of important parasitic infections including malaria, babesiosis, leishmaniasis, cryptosporidiosis, and toxoplasmosis [14]. Indeed, these compounds can exhibit a direct antiparasitic activity against the parasites, or an indirect effect, by exerting immunomodulating functions [24].

In this work, the monovalent ionophores exhibited good activities against promastigotes of all *Leishmania* spp. tested, inhibiting parasite growth at or below the micromolar range. This is an important finding since more than 20 species of *Leishmania* can infect humans, and several studies have reported different sensitivities of *Leishmania* species to different drugs with remarkable implications for clinical outcome [25,26]. Of the different ionophores tested, monensin and nigericin showed significant antiparasitic activities against extracellular promastigotes, with IC_50_s lower than 0.5 µM in some cases, and in turn not so different from that of Amphotericin B, used here as an anti-*Leishmania* reference compound. On the contrary, salinomycin was the least potent compound with an IC_50_ of around 5 µM against promastigotes of *L. tropica* and *L. braziliensis,* and ~8 µM against *L. infantum*. These results vary from what was reported in a previous work where the IC_50_ of salinomycin against promastigotes of *L. donovani* was around 1.7 µM [16]. The mechanism of action of salinomycin seems to be related to its ability to create an osmotic unbalance and subsequent swelling of organelle membranes [16]. Indeed, monensin also performed better than salinomycin against *Trypanosoma brucei* and *P. falciparum* [15,27]. Monensin seems to possess a huge therapeutic potential as antibacterial, antifungal, antiviral, and antiparasitic agent [28]. In an ex vivo splenic explant model of *L. donovani* infection, monensin showed an IC_50_ of 0.85 µM against intracellular amastigotes [17]. The present work reports a similar activity of monensin against *L. infantum* amastigotes using human differentiated THP-1 cells. In line with these broad-spectrum microbicidal effects, nigericin is also known to possess wide biological activities against bacteria, fungi, and malaria parasites. Here, we showed for the first time that nigericin exerts antiparasitic activity against promastigotes and amastigotes of *L. infantum* with a potency similar to that of monensin. Of note, all ionophores showed low in vitro cytotoxicity against human cells with high SI.

In this study, the ability of two different *Leishmania* species to infect human dTHP-1 and primary canine macrophages was also investigated. It was observed that both *L. infantum* and *L. tropica* promastigotes were phagocytosed by canine macrophages at high infection rates. In particular, the percentage of infected canine macrophages was higher than 85%, whereas the proportion of infected human dTHP-1 was about 25%. This was not completely unexpected, since it has been reported that macrophages of human, murine, or canine derivation exhibit different susceptibilities to *L. infantum* infections [29]. The reasons for such a behavior are not clear, yet. One possibility is that the membrane receptors involved in parasite recognition are different depending on the cell origin, their degree of differentiation, or the species analyzed. The receptors involved in *Leishmania* internalization include the first and the third complement receptors (CR1, CR3), the mannose receptor (MR), the Fc gamma receptors, and the fibronectin receptors (FnRs) [30]. It has been reported that THP-1 cells express CR1, CR3, and Fc gamma receptor, but not MR, also known as CD206 [31]. In contrast, canine monocyte-derived macrophages also express CD206 [32], which may contribute to the elevated infection rate observed. Furthermore, since canine macrophages seem to be unable to control parasite growth, the possibility that proliferating parasites were also capable of infecting new cells during the 72-h infection period cannot be ruled out.

Interestingly, monovalent ionophores were more cytotoxic in vitro against canine than human macrophages. This agrees with several studies that investigated the toxicity of ionophores after oral administration in different animal species. Cardiotoxicity, neurotoxicity and muscle degeneration by monensin have been widely described in different animal species, including dogs [33]. Based on oral toxicity data, it seems that in the same species, monensin is more toxic than salinomycin [34]. However, ionophore toxicity is often due to accidental overdose, being generally safe when used at the recommended dosage [35].

Of potentially clinical relevance is the fact that ionophores appeared to be less effective against *L. infantum* amastigotes within canine macrophages compared to human macrophages. In fact, drug treatment in dogs is often unable to clear the parasite burden, leading to a transient clinical cure and disease recurrence. In contrast, in immunocompetent human patients, drug treatment leads to complete cure at clinical and parasitological levels [36]. Furthermore, regardless of the drug of choice in the treatment of canine leishmaniasis, relapse often occurs, and treatment is often used to improve the quality of life of the dog [7]. The different activity of the ionophores against amastigotes inside canine vs. human cells could also be ascribed to their immunomodulatory properties. Nigericin is a well-known activator of the inflammasome (a multiprotein intracellular complex of the innate immunity) [37], which could contribute to the antiparasitic mechanisms reported here. However, the inflammasome activation in canine or human macrophages may be different, since a downregulation of the pathogen-sensing inflammasome pathways has been described in Carnivora, including canids [38].

Furthermore, salinomycin and the other ionophores are known to kill cancer stem cells through different mechanisms, which include the modulation of signaling pathways, the initiation of autophagy, mitochondrial dysfunction, oxidative stress, and apoptosis [39]. Thus, it is possible that one or more of these mechanisms might also be involved in the observed anti-*Leishmania* activity. By inducing apoptosis, the ionophores may counterbalance one of the strategies put in place by the parasites to persist within host cells [40]. In addition, another subversion mechanism employed by *Leishmania* spp. is the manipulation of oxygen radical (ROS) generation, a key microbicidal function by macrophages [41]. It is indeed well known that the currently used antileishmanial drugs exert their antiparasitic effects by generating ROS and by inducing modifications of the plasma membrane permeability. At present, there are no studies comparing the respiratory burst of human vs. canine macrophages. In fact, whether the different leishmanicidal abilities of ionophore-treated canine cells compared to human cells is due to a differential ROS generation or distinct antioxidant responses of the two species cannot be completely excluded.

## 5. Conclusions

The development of new antileishmanial compounds with improved safety profiles in humans and dogs is urgently needed. The present in vitro data suggest a potential application of monovalent ionophores against human leishmaniasis. Even if more studies are still required to investigate their in vivo efficacy and toxicity profiles, the process may be accelerated since they are repurposed drugs with known activity against a wide range of biological targets. These data may also provide valuable inputs for future drug optimization studies aiming to develop more effective and less toxic derivatives. Finally, considering the different activity of the ionophores against amastigotes in human and canine macrophages, this study emphasizes the need of further investments to identify new drugs with optimal therapeutic profiles to treat human and/or canine leishmaniases.

## Figures and Tables

**Table 1:** IC_50_ values of salinomycin, monensin and nigericin against *L. infantum*, *L. braziliensis*, and *L. tropica* promastigotes.

	IC_50_ (µM ± SD)		
	*L. infantum*	*L. braziliensis*	*L. tropica*
Salinomycin	7.98 ± 0.64	5.08 ± 1.74	4.53 ± 0.60
Monensin	0.63 ± 0.11	0.27 ± 0.13	0.35 ± 0.17
Nigericin	0.37 ± 0.04	0.78 ± 0.08	0.23 ± 0.05
Amphotericin B	0.12 ± 0.02	0.08 ± 0.02	0.10 ± 0.02

Data are the mean IC_50_ (µM) ± SD of three different experiments in duplicate. Parasite viability was measured by the MTT assay after 72 h of treatment.

**Table 2:** Cytotoxicity of salinomycin, monensin, and nigericin to dTHP-1 and HDF.

	CC_50_ (μM ± SD)	
	dTHP-1	HDF
Salinomycin	32.1 ± 5.3	>60
Monensin	>60	>60
Nigericin	>60	>60
Amphotericin B	>20	ND

ND = not determined. Data are from three independent experiments performed in duplicate. Cell viability was measured by the MTT assay after 72 h of treatment.

**Table 3:** Antiparasitic activity against *L. infantum* intracellular amastigotes and selectivity index of ionophores.

	Amastigotes IC_50_ (μM ± SD)	SI
Salinomycin	1.93 ± 0.1	16.6
Monensin	1.67 ± 0.43	>35.9
Nigericin	1.79 ± 0.83	>33.5
Amphotericin B	0.18 ± 0.03	>111.1

SI = Selectivity index = IC_50_ dTHP-1/IC_50_ amastigotes; data are the mean of IC_50_ (µM) ± SD of three different experiments in duplicate. dTHP-1 cells infected with *L. infantum* were treated with ionophores for 72 h. Infected cells were stained with Giemsa, and the percentage of inhibition was calculated in respect of infected and untreated dTHP-1 cells used as a positive control.

## Data Availability

Not applicable.
